# A systematic review and meta-analysis regarding the use of corticosteroids in septic arthritis

**DOI:** 10.1186/s12891-015-0702-3

**Published:** 2015-09-05

**Authors:** Luke Farrow

**Affiliations:** College of Medical, Veterinary and Life Sciences, University of Glasgow, Glasgow, UK; College of Medical, Veterinary and Life Sciences, Wolfson Link Building, University of Glasgow Glasgow, Scotland, G12 8QQ UK

## Abstract

**Background:**

Chondral damage is one of the major sequelae of septic arthritis; occurring even after prompt treatment of a septic joint. Subsequent loss of joint function can have a significant impact on a patient’s quality of life. Corticosteroids are known to have beneficial effects on the rate and extent cartilage destruction in arthritis through a variety of mediators such as synovial RANKL expression, mast cells and pro-inflammatory cytokines. Investigation into sepsis at other sites has suggested improved outcomes with corticosteroid use despite the theoretical risks. This study therefore set out to review current literature with regards to a possible beneficial effect for corticosteroids in Septic Arthritis.

**Methods:**

A computerised search of the databases MEDLINE and CINAHL was conducted during November 2014 using the EBSCOhost web search engine in order to identify research articles relating to the use of corticosteroids in the treatment of septic arthritis. The search strategy revealed 223 unique articles which were subjected to inclusion/exclusion criteria assessment. 6 articles were selected for study inclusion. These consisted of 3 human studies (2 double-blind randomised controlled trials & 1 double-blind non-randomised controlled trial), and 3 animal studies (3 non-blinded non-randomised controlled trials). Quantitative synthesis (meta-analysis) was only possible regarding two primary outcomes for two of the included studies – time to normalisation of CRP and duration of IV antibiotic therapy.

**Results:**

All current published evidence in humans is focused upon children. Overall results did however reveal a consensus between these studies for a reduced duration of symptoms and a reduction in inflammatory markers. Animal data suggested a protective effect on the articular cartilage with the addition of corticosteroids to antibiotic therapy. No article noted an adverse effect associated with steroid use. Findings were consistent with systematic reviews of corticosteroid use in other bacterial infections.

**Conclusions:**

Despite the promising outlook, issues’ regarding generalisability of results and a lack of large randomised controlled trial data necessitates further assessment of the safety and efficacy of steroid use in adults before treatment recommendations can be made. Long term safety data and the determinations of the optimum route, dose and timing of corticosteroids are also required.

## Background

Despite modern antibiotic therapy Septic Arthritis still carries a significant associated morbidity (31.6 %) and mortality (11.5 %) [1]. The higher incidence of infection in those of a younger age enhances the magnitude of this issue. Joint contamination typically occurs through haematogenous spread but direct inoculation can occur with intra-articular procedures. Clinical presentation is usually of a monoarthritis with systemic sepsis. The knee is most commonly affected, accounting for approximately 50 % of cases [2].

A vast array of pathogenic organisms have been identified; with gram positive cocci such as Staphylococcus and Streptococcus are the most commonly seen [3]. Once colonisation has occurred rapid proliferation of bacteria ensues initiating an acute inflammatory response. There is activation of Toll-like receptors with substantial release of pro-inflammatory cytokines such as Interleukin (IL)-1 beta, Tumour Necrosis Factor (TNF)-alpha, IL-17 and IL-6 [3]. These molecules stimulate osteoclast differentiation and bone reabsorption as well as matrix metalloproteinase (MMP) release leading to subsequent bone and cartilage degradation [4]. Presence of a joint effusion secondary to increased synovial fluid production increases intra-articular pressure which mechanically impedes blood/nutrient supply to the joint and further exacerbates cellular damage [2]. There is rapid progression and necrosis/degradation of chondrocytes within the first 24 h of onset [5, 6].

Corticosteroids are one well-established method of reducing joint inflammation, having being used in rheumatoid arthritis for decades [7]. Pharmacodynamic effects are mediated through binding to glucocorticoid receptors; stimulating targeted protein upregulation (in particular Lipocortin). There is subsequent inhibition of Phospholipase A2 (a potent intracellular producer of prostaglandins, free radicals and leukotrienes) as well as a deleterious effect on several pro-inflammatory cytokines such as IL-1, Interferon (IFN)-γ and TNF-alpha. Other functions include inhibition of elastase and collagenase production, decreased T-cell number and reduced synovial MMP-1 expression [8].

The use of glucocorticoids in infection has classically been contra-indicated due to a theoretical risk of worsening sepsis. Recent evidence regarding the use of corticosteroids alongside antibiotics in infection at other sites has however brought this suggestion into question. Studies into severe sepsis [9], severe pneumonia [10], bacterial meningitis [11] and acute pyelonephritis [12] have all shown improved outcomes associated with steroid use.

Current research therefore suggests evidence for both a positive effect of glucocorticoids on joint destruction in arthritis, and improved clinical outcomes in infection when combined with antibiotic therapy. It is hypothesised that the combination of corticosteroids and antibiotics could be of benefit when considering Septic Arthritis. This study therefore sets out to examine the available evidence on this research topic in the form of a systematic review and meta-analysis.

### Review

## Methods

A computerised search of the databases MEDLINE and CINAHL was conducted during November 2014 using the EBSCOhost web search engine. This identified research articles relating to the use of corticosteroids in the treatment of septic arthritis. Search criteria included combinations of the terms “steroids”, “hydrocortisone”, “corticosteroids”, “dexamethasone”, “triamcinolone”, “prednisolone”, “glucocorticoids”, “septic arthritis” and “joint infection” via a Boolean/Phrase strategy applied to the full text article and abstract. An outline of the full electronic search strategy is shown in Table 1.

**Table 1:** Search Criteria

Search 1: Steroids OR Corticosteroids OR Hydrocortisone OR Dexamethasone OR Triamcinolone OR Prednisolone OR Glucocorticoids (Search mode – Boolean/Phrase: 333,378 results).	
Search 2: Septic Arthritis OR Joint Infection (Search mode – Boolean/Phrase, 7661 results)	
Search 3: Search 1 AND Search 2 (Search mode – Boolean/Phrase: 223 results)	

Search restrictions included English language text with a publication date 1963–2014. Further potentially relevant literature was identified through bibliographical analysis of identified articles and using an internet search engine (Google). Initial screening of potential articles was performed through an independent abstract review. Relevant articles were then discerned from those initially identified with a thorough independent full text analysis. Inclusion and exclusion criteria were used to ensure any conclusions drawn from the literature were valid and based on a sound methodical construct. Inclusion criteria comprised: interventional articles relating to the concomitant use of corticosteroids and antibiotics in the treatment of septic arthritis; presence of a control group or cohort; clearly stated inclusion and outcome criteria; assessment of results using a validated appropriate statistical test and ethical approval. Exclusion criteria included: No full text availability and review articles.

Quality assessment was performed via the Cochrane Collaboration’s risk of bias tool. No formal review protocol was produced prior to the assessment process. All research was conducted in accordance with current international ethical standards [13].

Data extraction was carried out via a manual independent assessment of each article. Variables which were sought included study design, study aim, sample size, subject demographics, intervention, primary outcome measures, results, and author’s conclusions. Meta-analysis of pooled outcome measures was performed where possible (≥2 homogeneous randomised controlled studies with at least one matched primary outcome variable) using the Cochrane collaboration’s RevMan 5.3 tool [14].

The written report has been produced in concordance with Preferred Reporting Items for Systematic Reviews and Meta-analyses (PRISMA) guidelines [15].

## Results

**Fig. 1:**
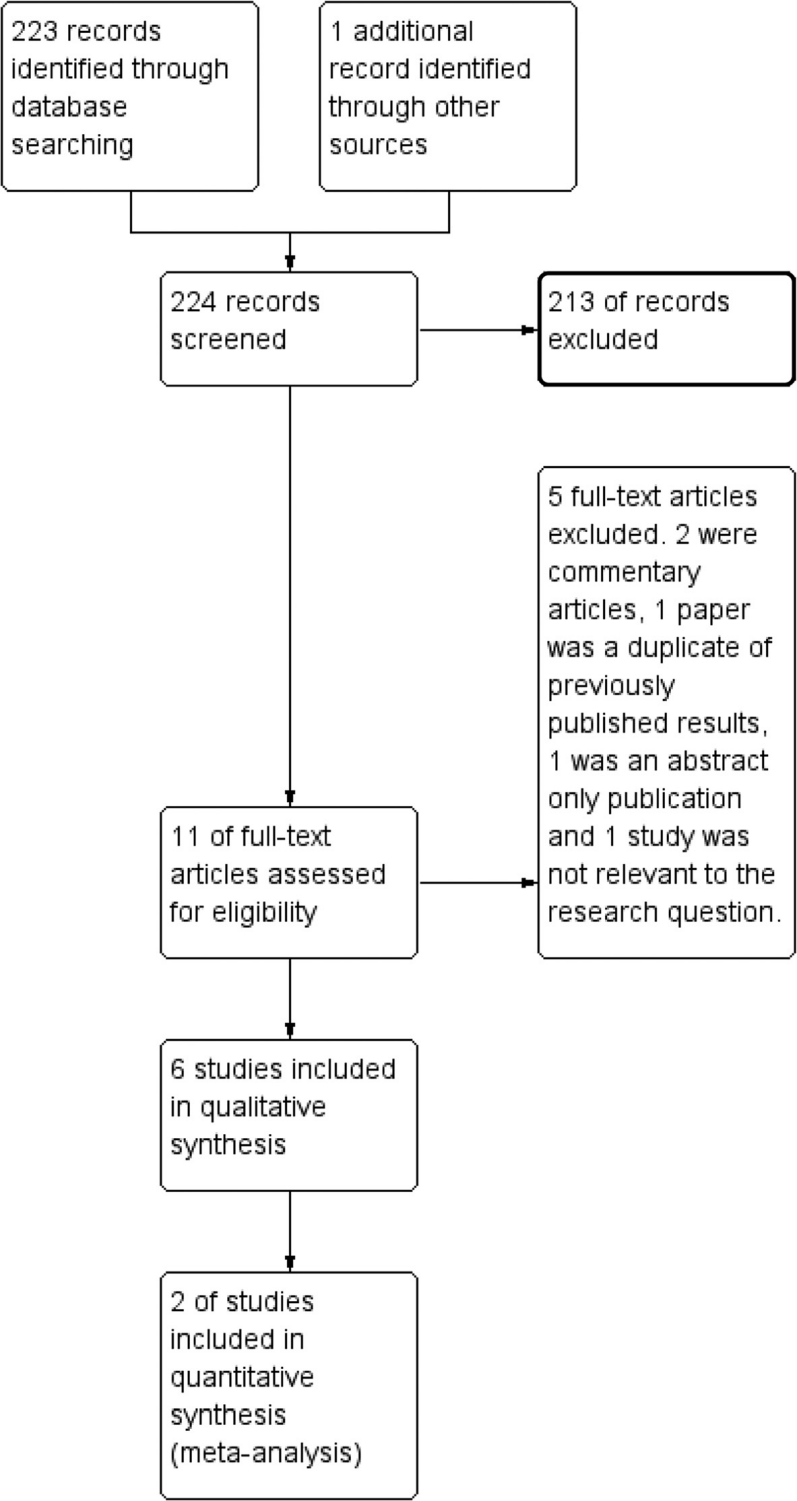
Flowchart diagram of the complete study selection process

The search strategy revealed 223 unique articles which were subjected to inclusion/exclusion criteria. Further abstract analysis identified 11 of these as potential candidates for review. A total of 6 articles were selected for study inclusion. A flowchart diagram of the complete study selection process is shown in Fig. 1. Articles consisted of 3 human studies in children (2 double-blind randomised controlled trials & 1 double-blind non-randomised controlled trial), and 3 animal studies (3 non-blinded non-randomised controlled trials). No interventional human adult studies were available for analysis. All studies showed a positive effect in at least one primary outcome for the addition of corticosteroids to antibiotics in the treatment of septic arthritis. A total of 349 participants were involved in the analysis (209 humans, 57 rabbits and 83 mice). The mean age of human participants was 5.64 years. Individual study characteristics and results are detailed in Table 2.

**Table 2:** Individual study characteristics and results

Authors	Design	Aims	Subjects	Intervention	Outcomes	Results
Stricker et al. 1996 [21]	Non-blinded non-randomised controlled trial	The chondroprotective effect of betamethasone was examined to determine if corticosteroids can decrease articular cartilage injury caused by inflammatory exudate in Staphylococcus aureus gonarthritis in rabbits	27 adolescent male New Zealand white rabbits split into 3 groups.	Each rabbit’s left knee was inoculated with Staphylococcus aureus. Group 1 received antibiotics alone (intramuscular Ceftriaxone for 12 days), group 2 received intramuscular corticosteroids (4 days Betamethasone) and antibiotics, group 3 received intra-articular glucocorticoids (single dose of Betamethasone) and antibiotics. All treatment was commenced at 36 h after joint inoculation.	Synovial fluid culture & cell count as well as peripheral leukocyte count at days 2, 6 and 14. Histological and histochemical analysis was undertaken at 14 days.	A significant difference was seen in mean peripheral leukocyte count when comparing group 1 and 2 at 14 days (5,100 vs 8,100 white blood cells, *p* < 0.05). Group 2 showed statistically greater safranin O stain orthochromasia than Group 1 or 3 (*p* < 0.05) which was thought to suggest prostaglandin preservation. This was consistent with biochemical results for hexosamine preservation when group 2 was compared to group 1 (*p* < 0.05)
Sakiniene et al. 1996 [23]	Non-blinded non-randomised controlled trial	To evaluate the combined effect of systemic corticosteroids and antibiotics on the course of septic arthritis	Two separate experimental protocols were used: 1–40 five week old Swiss mice (20 male, 20 female) split into 3 groups. 2 – 43 male Swiss nice (5–6 weeks old) split into 3 groups. Results of these experiments were pooled.	In both studies mice were initially inoculated with Staphylococcus aureus. One group received no treatment, one group received antibiotics alone (Cloxacillin given intraperitoneally every 12 h) and one group received corticosteroids (Dexamethasone given intraperitoneally every 24 h) and antibiotics. Intervention was commenced at 3 days post inoculation.	Frequency and severity of arthritis (Arthritis index) at 0,3,7,14 days. Histopathological, immunohistochemical, bacteriologic, serologic and haematologic manifestations were assessed at 14 days.	Only those treated with antibiotics and corticosteroids showed a significantly difference in the frequency and severity of arthritis compared to controls at 14 days. Serum levels of IFN-γ were 4 fold decreased in the antibiotic treated group and 15 fold decreased in the corticosteroid and antibiotic group. Serum NO3^−^ levels were significantly reduced in both the treatment arms.
Wysenbeek et al. 1998 [24]	Non-blinded non-randomised controlled trial	To assess the effect of intra-articular corticosteroids added to systemic antibiotics in experimental septic arthritis.	30 eight week old New Zealand rabbits split into 3 groups (*n* = 10 per group).	Rabbits had their left knee inoculated with Staphylococcus epidermis. Group 1 were untreated controls, group 2 were treated with antibiotics alone (daily intramuscular Cefonicid) and group 3 received glucocorticoids (intra-articular Methylprednisolone given 48 h after infection and 24 h after antibiotic commencement) and antibiotics	A composite score of Histopathological-histochemical parameters in both a vertical (A) and horizontal (B) section at follow up (15 days).	Both treatment groups had significantly lower scores than the control. Group 3 had significantly lower scores than group 2 for A (6.5 [1.4] vs 4.0 [1.0], *p* = 0.001) and B (7.4 [2.6] vs 4.2 [2.2], *p* = 0.01) [] = Standard Deviation (SD).
Odio et al. 2003 [17]	Double-blind randomised controlled trial	Assess the impact of corticosteroids and antibiotics on joint dysfunction and the acute response in septic arthritis.	123 children with documented septic arthritis aged 3 months to 11 years (with parental consent). 23 children were unevaluable therefore 100 patients were assessed. There were two groups: Dexamethasone (*n* = 50) and Placebo (*n* = 50). There were no significant differences in baselines characteristics between groups	Placebo group received antibiotics and IV 0.9 % saline (equivalent of Dexamethasone timing and volume). There was a variety of antibiotic regimes used based on age, identified pathogen and organism resistance. Dexamethasone group received antibiotics and IV Dexamethasone (0.2 mg/kg/dose given every 12 h for 8 doses). Dexamethasone/Placebo given from diagnosis.	Clinical outcomes were assessed at the end of treatment, 6 months and 12 months. During the acute phase time for normalisation of CRP, duration of symptoms, extent of antibiotic therapy and arthrotomy requirements were also assessed.	Levels of residual dysfunction were significantly improved for Dexamethasone vs. Placebo at end of treatment (2 vs 16 [5.54–16.62], *p* < 0.001), at 6 months (1 vs 19 [1.41–4.22], *p* < 0.001) and 12 months (1 vs 13 [1.31–4.1], *p* < 0.001) [] = 95 % CI. Compared to placebo the Dexamethasone group also had quicker normalisation of CRP (2.04 ± 1.25 vs 4.68 ± 6.23 days, *p* = 0.01); earlier resolution of symptoms (2.34 ± 5.06 vs 7.81 ± 2.04 days, *p* = 0.001); and shorter duration of IV antibiotics (7.2 ± 1.2 vs 10 ± 5.6, *p* < 0.05). ± = SD
Harel et al. 2011 [16]	Double-blind randomised controlled trial	Evaluate the effect of adding dexamethasone to antibiotic therapy in the clinical course of septic arthritis in children.	49 children aged 6–161 months with confirmed septic arthritis. There were two groups: Dexamethasone (*n* = 24) and Placebo (*n* = 25)	Placebo group received antibiotics (IV Cefuroxime 150 mg/kg/day in 3 divided doses) and placebo (IV 0.9 % saline at equivalent of Dexamethasone timing and volume). Dexamethasone group received antibiotics and IV Dexamethasone (0.15 mg/kg/dose) every 6 h for 16 consecutive doses. Dexamethasone/Placebo given from diagnosis.	Comparison between groups for clinical and laboratory parameters, length of hospital stay, and late sequelae (follow up at 2,6,12 months).	Compared to placebo those treated with Dexamethasone had a shorter duration of fever (1.68 [2.57] vs 2.38 [2.41] for first day without fever, *p* = 0.021); quicker normalisation of CRP (3.09 [3.80] vs 5.48 [4.69] days, *p* = 0.029); and duration of IV antibiotics (9.91 [4.84] vs 12.60 [5.20] days, *p* = 0.007). No formal assessment of late sequelae or length of hospital stay was performed. [] = SD
Arti et al. 2014 [18]	Double-blind non-randomized controlled trial	Evaluation of the effects of IV dexamethasone on the recovery process in septic arthritis	60 patients with septic arthritis separated into two groups (*n* = 30). Mean age in group 1 was 8.06 ± 0.5 years. 73 % of the group 1 participants were male and 27 % were female. Mean age in group 2 was 8 ± 0.6 years. 70 % of the group 1 participants were male and 30 % were female.	Group 1 received IV antibiotics (dose & timing not detailed) and IV Dexamethasone (0.15 mg/kg/dose every 6 h for 4 days). Group 2 received antibiotics and placebo (IV 0.9 % saline given at same volume and timing as Dexamethasone). Dexamethasone/Placebo given from diagnosis.	Groups were compared regarding clinical (inflammation & redness, range of motion (ROM), and duration of therapy) and laboratory findings (ESR & CRP).	There was a significant difference between groups 1 & 2 regarding redness & inflammation (2.43 ± 0.15 vs 6.53 ± 0.27 days, *p* = 0.0014); days of hospitalisation (8.9 ± 0.88 vs 12.17 ± 0.54 days, *p* = 0.005); joint ROM (91.2 ± 2.28 vs 40.53 ± 1.44°, *p* = 0.008); and reduction of ESR levels (36.03 ± 2.77 vs 16.73 ± 0.83, *p* = 0.0001) ± = SD

**Fig. 2:**
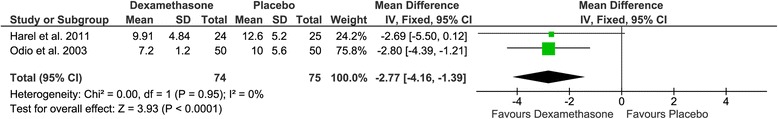
Forest plot – meta-analysis of days to normalisation of CRP for Odio et al. 2003 & Harel et al. 2011

**Fig. 3:**
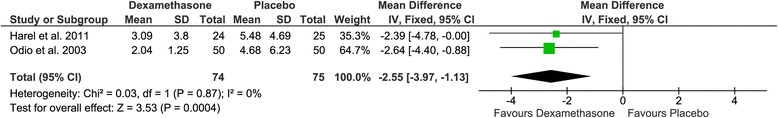
Forest plot – meta-analysis of duration of IV antibiotics for Odio et al. 2003 & Harel et al. 2011

**Fig. 4:**
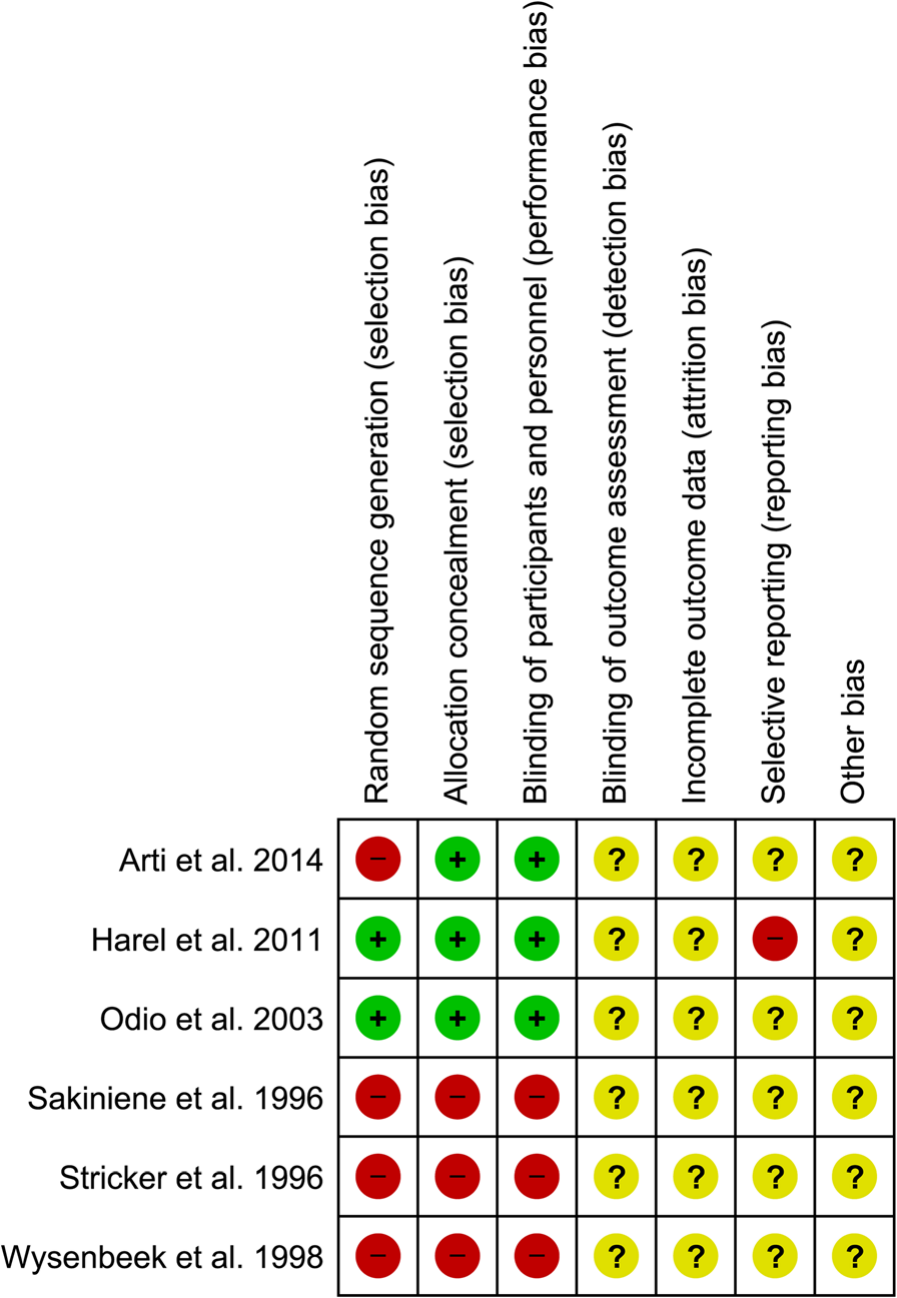
A risk of bias table detailing the author’s judgements about each risk of bias item for each included study

Quantitative synthesis (meta-analysis) was only possible regarding two primary outcomes for two of the included studies – time to normalisation of CRP (Fig. 2) and duration of intra-venous (IV) antibiotic therapy (Fig. 3). A risk of bias table detailing the author’s judgements about each risk of bias item for each included study is shown in Fig. 4.

## Discussion

There was a significant lack of adult human interventional trial data available. All studies did however show a positive effect in at least one primary outcome for the addition of corticosteroids to antibiotics in the treatment of septic arthritis. These improvements were shown across a variety of cellular, histological and clinical levels. Meta-analysis regarding normalisation of CRP and duration of IV antibiotics was performed with statistically significant trends favouring the addition of Dexamethasone vs. placebo for both. A lack of available studies and participant numbers to provide substantial analysis were however noted. No study documented adverse events associated with glucocorticoid use. These results are in keeping with those examining the addition of corticosteroids to antibiotics at other anatomical sites. Whilst these results are promising, there is a lack of large randomised controlled trial evidence with long term follow up and safety data in adult human subject’s. As such there is currently insufficient evidence to recommend corticosteroids alongside antibiotics for the treatment of septic arthritis.

The studies identified during the review process were of varying methodological quality. There were two double-blind randomised controlled trials identified during the search process [16, 17]. Both of these used a placebo controlled design to provide evidence for a positive effect in both biological and clinical parameters for dexamethasone in addition to antibiotic therapy. The numbers involved were however small, and there were some flaws identified in the methodological design of both studies. For example both articles failed to document if there was blinding of outcome assessment; determine whether appraisal was performed on an intention-to-treat or per-protocol; perform any formal safety analysis; and account for the impact of surgical intervention on outcomes. In addition despite documentation of duration of hospitalisation as a primary outcome in Harel et al. 2011 [16] the results and statistical analysis were not included in the published article. This study also failed to perform any statistical assessment of long term outcomes – possibly due to significant participant attrition at 12 months follow up.

The other human study by Arti et al. 2014 [18] also found a positive effect for Dexamethasone therapy alongside antibiotics with regards to number of days of hospitalisation, clinical outcomes, and laboratory findings. This study had a number of major methodological flaws such as lack of randomisation, failure to compare baseline characteristics between groups and no long term follow up. It was therefore felt to provide minimal additional information to the previous literature.

The remaining studies focused upon the use of corticosteroids alongside antibiotics in an animal model. These allowed for direct analysis of histopathological parameters not easily assessed in humans due to ethical concerns. All studies showed an improvement in arthritis severity with the addition of glucocorticoids to antibiotics. Despite the poor methodological quality of the research and the lack of direct applicability to humans these articles do provide valid scientific rationale for the use of corticosteroids in septic arthritis.

There are still however a number of unanswered questions regarding the use of corticosteroids in Septic Arthritis. Importantly the concerns coming from a previously identified association between the use of glucocorticoids and an increased risk of infection [19] have not yet been fully addressed. Although no study assessed during this analysis identified any safety issues during follow up this was not formally evaluated at any point. A recently published abstract did report safety during a retrospective analysis regarding the use of corticosteroids in septic arthritis with no side effects noted except for 3 cases of symptom rebound after completion of the steroid course. This is a previously established side effect of steroid withdrawal [20]. It is also important to consider the significant long term effects of steroid use such as osteoporosis, diabetes, raised blood pressure, weight gain, mood changes and gastric irritation. It is felt however that the duration and dose of corticosteroids used in this scenario (such as the 4 days of IV Dexamethasone used in Odio et al. 2003 [17] and Harel et al. 2011 [16]) would be unlikely to have any significant long term effect. The optimum route, duration and dose of glucocorticoids need to be determined as well. Comparisons between intramuscular and intra-articular corticosteroids in the Stricker et al. 1996 [21] study did appear to favour an increased effect with the parenteral route. No study has examined the oral route of administration.

This study has also highlighted issues regarding the generalisability of current evidence. So far all studies have focused upon a child population and therefore the impact on and adult cohort is still unknown. It can be hypothesised that known differences in immunology between varying age groups [22] could impact on the glucocorticoid response.

## Conclusions

The current evidence base points strongly towards a beneficial effect for corticosteroids in septic arthritis. This is consistent with scientific rationale and data regarding glucocorticoid use in sepsis at other anatomical sites. There is however insufficient evidence to make treatment recommendations at present. Further large scale randomised controlled trials including adult subjects; safety profiles and long term efficacy are required for this promising therapeutic option.
